# Early-life exposure to Chinese famine and stroke risk in mid- to late life: the mediating roles of cognitive function and depression

**DOI:** 10.1186/s12877-022-02990-z

**Published:** 2022-04-07

**Authors:** Zi Zhou, Wei Zhang, Ya Fang

**Affiliations:** 1 State Key Laboratory of Molecular Vaccinology and Molecular Diagnostics, School of Public Affairs and School of Public Health, Xiamen University, Xiamen, 361102 Fujian China; 2 Key Laboratory of Health Technology Assessment of Fujian Province University, School of Public Health, Xiamen University, Xiang’an South Road, Xiamen, 361102 Fujian China

**Keywords:** Famine exposure, Stroke, Marginal structural models, Mediating analysis

## Abstract

**Background:**

Limited research has examined the role that famine exposure plays in adulthood stroke risk. We aim to explore the causal implications of early exposure to the Great Chinese Famine on stroke risk and determine whether these associations were mediated by cognitive function, and depression.

**Methods:**

We sampled 12,681 individuals aged 45 years and older from the China Health and Retirement Longitudinal Study (CHARLS) and divided them into fetally exposed, childhood-exposed, adolescence/adulthood-exposed and unexposed groups. Stroke was defined by self- or proxy-reported physician diagnosis. Based on a counterfactual framework, marginal structural models were used to estimate the natural direct effect and the natural indirect effects through cognitive function and depression for causal inference.

**Results:**

We found that early-life exposure to Chinese famine was directly related to increased stroke risk in mid- to late life. Cognitive function and depression accounted for a greater part of the effect for childhood famine exposure, mediating 36.35% (95%CI: 14.19, 96.19%) of the overall association between famine exposure and incident stroke, than for the fetal, adolescence/adulthood famine exposure groups. However, the natural indirect effect through depression was not significant in the fetally exposed group. The results were robust in the sensitivity analysis of model specification and unobserved confounding.

**Conclusions:**

Our findings are consistent with the latency, pathway, and accumulation models, supporting the life-course theory. Early stages of life exposed to the Chinese Famine were associated with higher risk of stroke in mid- to late life. Enhanced cognitive and depression interventions may reduce stroke risk in middle-aged and older Chinese adults who exposure to famine in early life.

**Supplementary Information:**

The online version contains supplementary material available at 10.1186/s12877-022-02990-z.

## Background

Stroke is the second leading cause of death worldwide [1] and the leading cause of death and disability-adjusted life-years (DALYs) lost in China. The absolute numbers and rates per 100,000 people for all­age DALYs lost to stroke increased by 46.8% between 1990 and 2017 [2]. Therefore, in the face of rapidly rising number of Chinese older adults, identifying risk factors for stroke might be the most viable avenue for lessening the burden of stroke on society. Previous work has focused on risk factors in adulthood, such as low cognitive function, depression, and hypertension, which are established targets for prevention but explain only part of the variance in stroke risk [3–5]. In addition to risk factors in adulthood, previous studies, adopting a life-course perspective, suggest that early life conditions (such as famine exposure) may affect later health [6–8]. The existing literature shows that early exposure to famine, an adverse situation characterized by malnutrition and stress, increased the risk of low cognitive function [9], depression [10], obesity [11], hypertension [12], and diabetes mellitus [13] in adult life.

However, limited research has examined the association between early exposure to famine and the risk of stroke in adulthood. The Dutch famine cohort study found a reduced stroke risk among women exposed to famine as children or young adults [14], whereas others suggest that prenatal exposure to famine does not increase the risk of adult stroke after controlling for stroke risk factors such as hypertension and obesity [15]. The findings are inconsistent, and mostly from the Dutch famine of 1944–45, focused only on prenatal exposure or studied only women. The Great Chinese Famine of 1959–61 was larger in terms of duration, geographical scope and level of damage than the Dutch famine. In China, people who have suffered from famine during fetal and postnatal life are generally over the age of 45. The Great Chinese Famine, with its widespread undernutrition and lack of food availability [16], serves as a natural experiment, providing a valuable opportunity to study the relationship between famine exposure at different developmental ages and stroke in the Chinese population.

Moreover, few studies have examined the causal pathway by which famine exposure affects stroke risk. The issue is crucial because understanding the causal basis of such associations is expected to improve our understanding of the health consequences of early life conditions and might have implications for practitioners and policymakers seeking targeted, population-specific intervention strategies to reduce stroke risk. The life-course theory proposes three conceptual models – the latency, pathway, and accumulation models – to explain potential causal pathways from early famine exposure to stroke in middle and older age [17, 18]. The latency model highlights the direct effect of early life conditions on later health status. The developmental origins of health and disease hypothesis proposes that early-life malnutrition may have a lasting impact on neurodevelopment and neuropsychiatric disorders in later life by damaging normal biological conditions permanently [19]. Early-life malnutrition, especially during fetal life and infancy, may also disrupt brain development and lead to neurodevelopmental anomalies [20].

The pathway model posits that conditions in early life may affect later health status indirectly via mental or psychological factors such as cognitive function and depression [6, 21]. For example, early exposure to famine can lead to low cognitive function [9, 22] and an increased prevalence of depression [10, 23] in adulthood, which are demonstrated to be important risk factors for stroke [3, 4, 24]. Thus, the relationship between famine exposure and stroke may be mediated by cognitive function and depression. The accumulation model focuses on the cumulative negative effect of adverse early life conditions across the life course, similar to a dose–response relationship. These three models are not mutually exclusive and can operate simultaneously. Moreover, it is difficult for conventional regression methods to evaluate the mediating effect of longitudinal data, as time-dependent covariates may create selection bias due to collider stratification, and the regression will ignore the situations where these variables might mediate the exposure effect [25].

The aim of our work was to explore the causal implications of early exposure to the Great Chinese Famine on stroke risk through a marginal structural model (MSM) using a longitudinal sample of nationally representative middle-aged and older Chinese adults. Since the timing and duration of the famine exposure varied by birth cohort, we compared stroke incidence across multiple birth cohorts. According to the latency model, we hypothesized that early famine exposure would increase the risk of stroke in adulthood. Guided by the pathway and accumulation models, we aimed to estimate whether the most common mental health conditions, low cognitive function and depression, partially mediate the associations between early famine exposure and stroke incidence in middle-aged and older Chinese populations.

## Methods

### Data and sample

We used data from the China Health and Retirement Longitudinal Study (CHARLS), a longitudinal, nationally representative study of middle-aged and older Chinese residents (≥45 years of age). The design of the CHARLS questionnaires was based on international experience, including the Health and Retirement Study (HRS) and the English Longitudinal Study of Ageing (ELSA), to ensure international comparability of results. The survey selected samples according to a multistage regional probability sampling design to create a high-quality public micro-database with extensive data from the Chinese population; this database contains detailed information about socioeconomic and demographic factors, family information, and health status. The original sample included 17,708 respondents who were selected randomly from 450 villages/resident committees, 150 counties/districts and 28 provinces with a probability proportional to scale (PPS) in 2011. In the case of home sampling, Google Earth (Google, Inc., Mountain View, California) was used to obtain photographs and GPS positions of all the buildings in each main sampling unit (PSU), and the homes in each building were listed. The respondents were followed up in a face-to-face computer-assisted personal interview every 2 years. In addition, CHARLS organized and implemented the “life history survey of Chinese residents” in 2014, including childhood history, residence and relocation history, health history, and other areas. The Ethical Review Committee of Beijing University reviewed and approved the CHARLS, and all participants read and signed an informed consent form before taking part in the CHARLS. Detailed information about sampling design and data quality management can be found elsewhere [26].

We chose 2011 as the baseline and included follow-up data from 2013 and 2015. We used data from Wave 3 (2014) of CHARLS to assess childhood covariates in China. We excluded respondents who reported a history of stroke (*n* = 413) at baseline and at the initial follow-up (2013). In addition, we also excluded participants who were missing important variables related to exposure (*n* = 774) and those died or were lost to follow-up between 2013 and 2015 (*n* = 3840). Of the respondents initially selected, 12,681 were eligible for further analysis. Each subject in this study was assigned a sampling weight, and our analysis used these weights to produce nationally representative results.

### Measures

#### Famine exposure and severity

The famine lasted from 1959 to 1962. Based on birth year and a previous Chinese famine study [27, 28], participants were divided into four groups according to when they were exposed to famine: never (1963–1966), during the fetal period (1959–1962), during childhood (1949–1958), or during adolescence or early adulthood (1921–1948). The Great Chinese Famine affected all provinces of China, but the severity of famine fluctuated sharply across regions due to the different provinces’ climates, population densities, and policies related to food shortages [29]. According to previous studies [30, 31], we used the excess death rate (EDR) as an indicator of the severity of the provincial famine. The EDR calculates the difference between the highest mortality rates during the famine (1959–1961) and the average level in 1956–1958, using a threshold of 100% excess mortality to distinguish participants born in less or more severely affected areas.

#### Stroke

Information on stroke was collected in the fourth-wave survey (2015), based on the following question: “Have you ever been diagnosed with stroke by a doctor?” Participants answering “Yes” were defined as having had a stroke, and participants answering “No” were defined as not having had a stroke.

#### Depression

The 10-item Center for Epidemiological Studies Depression Scale (CES-D-10) was used to measure depression [32]. Each item was evaluated on a 4-level Likert scale, from “rarely or none of the time (< 1 day)” to “most or all of the time (5–7 days)”, scored as 0 to 3 points, for a total score of 0 to 30. The cutoff point of 12 has been demonstrated to be an optimal threshold to identify whether elderly respondents in China suffer from depression [33]; this cutoff value achieves good sensitivity (0.76) and specificity (0.55) [34]. Depression was evaluated at the initial follow-up (2013) visit as a mediator.

#### Cognitive function

We constructed cognitive scores based on similar measures used in other CHARLS studies [35]. Episodic memory was the mean score calculated for immediate and delayed recall of 10 words. The Telephone Interview of Cognitive Status (TICS) battery has come to be used to examine the intactness of individuals’ mental status. This battery includes counting down from 100 in steps of 7 for 5 iterations; naming the day of the week and the current date (day, month, year and season); and redrawing a picture from memory. We converted the answers to these questions to a mental status score, ranging from 0 to 11. General cognition was measured using the total scores for the two measures above, ranging from 0 to 21. Cognitive function was evaluated at the initial follow-up visit (2013) as a mediator.

#### Other covariates

In this study, potential covariates included time-constant and time-varying covariates. Time-constant covariates including sex (male or female), ethnicity (Han or minority), residence (rural or urban) and self-reported childhood economic status and health status (good, fair or poor) were measured at the baseline wave and the third wave. Time-varying covariates, which were measured at the baseline wave (2011) and the initial follow-up (2013), included age (year), marital status (currently married or not married), body mass index (≥24 or < 24 kg/m^2^), educational level (lower than primary school or primary school and above); chronic diseases included hypertension, dyslipidemia, and diabetes, which were dichotomized as yes (1) or no (0). Smoking status and drinking status were categorized as current (2), former (1), or never (0). Income was measured by the continuous variable of household per capita expenditure (PCE) in the past year; this variable is a better economic status measure than income in developing countries, since most rural residents have no regular income [36]. PCE was log transformed because of the skewness of its distribution.

### Statistical analyses

Descriptive statistics were used to summarize the characteristics of the baseline sample (2011) by using distribution ratios, means and deviations. We used analysis of variance (ANOVA) and *χ*^2^ tests to test the differences in continuous variables and categorical variables, respectively.

**Fig. 1:**
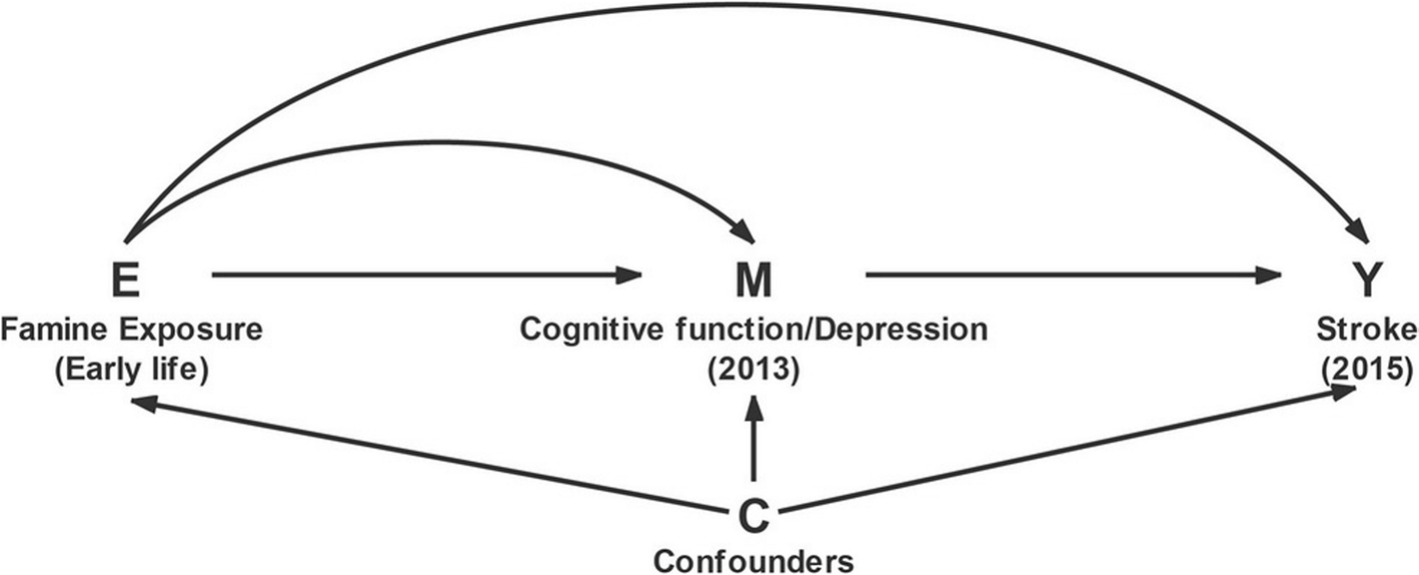
Hypothetical Relationship among Famine Exposure, Cognitive Function and Depression, Stroke Incidence, and Confounders in CHARLS, 2011–2015

We used MSM by VanderWeele [37] to estimate the natural direct effect (NDE) and natural indirect effect (NIE). The model was fitted in a two-stage process. First, inverse probability weights were used to handle the confounders in the MSM. Each observation of the individual was weighted using an inverse-probability-of treatment weight (IPTW). We estimated the weights of exposure and mediators separately and then multiplied them to obtain the final weight. To adjust for censoring, we calculated an inverse-probability-of-censoring weight in MSM. Each participant in this study was assigned a sampling weight (${\mathrm{SW}}_i^S$) in the CHARLS database, and our analysis also used these weights to produce nationally representative results. Second, we used 2 MSMs to estimate the NDE and NIE. For further details on MSM analysis, please see Additional file 1. Figure 1 shows a hypothetical relationship between famine exposure and stroke risk.

To evaluate whether the associations between famine and stroke risk were dose-dependent, we classified participants according to the joint categories of when they were exposed to famine and the severity of the famine. We used those unexposed to famine as a reference group.

We performed a series of sensitivity analyses to evaluate the robustness of MSM to extremum of weights and unmeasured confounder (*U*). First, we used another two weight truncations (5th/95th and 10th/90th percentiles) to evaluate the associations. Second, we used a method by VanderWeele [38] to estimate NDE and NIE, assuming the existence of an unmeasured binary confounder (*U*). We defined the sensitivity parameter g as the effect of *U* on stroke and *d* as the difference in prevalence confounder *U* between levels of exposure or mediators. The values of the parameters *g* and *d* were set according to the relevant meta-analysis [4, 39]. We assumed g values of ±0.4 and d values of ±0.3. Each possible product of *g* × *d* was then subtracted from the *β* coefficient (the NDE and NIE) and 95% CI derived from the MSM under each confounding specification. If the sensitivity analyses revealed that most of the *β* coefficients remained similar, than the unmeasured confounding effects were ignored [40, 41].

The multiple imputation by chained equations (MICE) procedure in Stata was used to replace the remaining missing data, assuming that the imputed data were missing at random. All statistical analysis was carried out with Stata version 16.0 (StataCorp; College Station, TX). Additional file 1 includes the sample Stata codes to create the dataset.

## Results

### Sample characteristics

Table 1 shows the baseline characteristics of the participants exposed to famine at different stages of life (unweighted). Overall, of the 12,681 adults, 1575 (12.42%) were from the fetally exposed group, 5129 (40.45%) were from the childhood-exposed group, and 4009 (31.61%) were from the adolescence/adulthood-exposed group. In the overall cohort, 245 persons (1.94%) reported having stroke. Compared with the unexposed cohort, those with exposure to famine had an increased likelihood of being male, unmarried, less educated, and current smokers and of suffering from hypertension, diabetes, dyslipidemia, depression and poor cognitive function. The proportion of alcohol consumption was higher in the fetal famine-exposed groups than in the other groups.

**Table 1:** Baseline Characteristics of Participants by Life Stage at the Time of Famine Exposure in CHARLS

	Unexposed 1963–1966 (*N* = 1968)	Fetally exposed 1959–1962 (*N* = 1575)	Childhood-exposed 1949–1958 (*N* = 5129)	Adolescence/adulthood-exposed 1921–1948 (*N* = 4009)	*P* value
**Childhood covariates, n (%)**					
Childhood economic status					0.153
Good	192 (9.76)	150 (9.52)	416 (8.11)	359 (8.95)	
Fair	1021 (51.88)	818 (51.94)	2616 (51.00)	2047 (51.06)	
Poor	755 (38.36)	607 (38.54)	2097 (40.89)	1603 (39.99)	
Childhood health status					0.248
Good	678 (34.45)	565 (35.87)	1815 (35.39)	1372 (34.22)	
Fair	1041 (52.90)	831 (52.76)	2619 (51.06)	2118 (52.83)	
Poor	249 (12.65)	179 (11.37)	695 (13.55)	519 (12.95)	
**Adulthood covariates, n (%)**					
Age in 2011^a^	46.76 (1.07)	50.29 (1.17)	57.54 (2.76)	69.84 (5.75)	< 0.001
Male	840 (42.68)	723 (45.90)	2505 (48.84)	2004 (49.99)	< 0.001
Rural residence	1221 (62.04)	997 (63.30)	3335 (65.02)	2597 (64.78)	0.085
Married	1912 (97.15)	1489 (94.54)	4744 (92.49)	3083 (76.90)	< 0.001
Ethnic Han	1823 (92.63)	1430 (90.79)	4720 (92.03)	3746 (93.44)	0.004
Education					< 0.001
Less than primary school	462 (23.48)	432 (27.43)	2620 (51.08)	2330 (58.12)	
Primary school or above	1506 (76.52)	1143 (72.57)	2509 (48.92)	1679 (41.88)	
Exposed to severe famine	792 (40.24)	579 (36.76)	2078 (40.51)	1662 (41.46)	0.015
Income (Chinese yuan)^a^	30,687.19 (41,771.75)	29,800.58 (48,565.44)	25,778.89 (38,968.84)	21,525.62 (36,813.99)	< 0.001
Smoking status					< 0.001
Nonsmoker	1308 (66.46)	984 (62.48)	2996 (58.41)	2333 (58.19)	
Former smoker	168 (8.54)	133 (8.44)	554 (10.80)	500 (12.47)	
Current smoker	492 (25.00)	458 (29.08)	1579 (30.79)	1176 (29.33)	
Drinking status					< 0.001
Nondrinker	1195 (60.72)	920 (58.41)	2956 (57.63)	2348 (58.57)	
Former drinker	85 (4.32)	82 (5.21)	379 (7.39)	463 (11.55)	
Current drinker	688 (34.96)	573 (36.38)	1794 (34.98)	1198 (29.88)	
Overweight	974 (49.49)	762 (48.38)	2119 (41.31)	1374 (34.27)	< 0.001
Hypertension	275 (13.97)	287 (18.22)	1138 (22.19)	1223 (30.51)	< 0.001
Dyslipidemia	127 (6.45)	138 (8.76)	459 (8.95)	385 (9.60)	0.001
Diabetes	60 (3.05)	81 (5.14)	290 (5.65)	238 (5.94)	< 0.001
Stroke	21 (1.07)	26 (1.65)	75 (1.46)	124 (3.09)	< 0.001
Depression	404 (20.53)	384 (24.38)	1411 (27.51)	1319 (32.90)	< 0.001
Cognitive function ^a^	13.15 (2.92)	12.67 (3.11)	11.85 (3.27)	10.73 (3.46)	< 0.001

^a^ The mean (standard deviation) was used to present the continuous variables

The NDE and NIE of early-life famine exposure on stroke as determined by mediation analysis are illustrated in Table 2. We first considered the mediating effect through cognitive function. The NIE of famine exposure through cognitive function varied by famine exposure stage, with the strongest NIE observed for those exposed to famine during adolescence/adulthood when compared with the unexposed group (NIE of 0.11; 95% CI = 0.01–0.20). When cognitive function and depression were combined, the NDE of different stages of famine exposure on stroke remained substantial. In the fetal famine-exposed group, only cognitive function emerged as a mediator of the effects of famine on stroke in adulthood (NIE of 0.02; 95% CI = 0.01–0.05). However, depression was the important mediator between childhood or adolescence/adulthood exposure to famine and stroke in mid- to late life, with NIE values of 0.28 (95% CI = 0.13–0.51) and 0.44 (95% CI = 0.22–0.70), respectively.

**Table 2:** Estimates of the Direct and Indirect effects of Famine Exposure on Stroke in CHARLS, 2011–2015

Mediators	Fetally exposed vs Unexposed		Childhood-exposed vs Unexposed		Adolescence/adulthood-exposed vs Unexposed	
	*β*	95% CI	*β*	95% CI	*β*	95% CI
Cognitive function (M_1_) ^a^						
NDE	0.75^*^	0.20, 1.57	0.64^*^	0.23, 1.29	1.50^*^	1.24, 2.16
NIE	0.02^*^	0.01, 0.04	0.06^*^	0.01, 0.10	0.11^*^	0.01, 0.20
Cognitive function (M_1_) and depression (M_2_) ^b^						
NDE	0.76^*^	0.26, 1.60	0.58^*^	0.02, 1.20	1.45^*^	1.00, 2.16
NIE through M_1_	0.02^*^	0.01, 0.05	0.05^*^	0.02, 0.11	0.11^*^	0.04, 0.21
NIE through M_2_	0.12	−0.03, 0.32	0.28^*^	0.13, 0.51	0.44^*^	0.22, 0.70

*CI* confidence interval, *NDE* natural direct effects, *NIE* natural indirect effects

^a^ Stabilized weights accounted for age, sex, ethnicity, type of residence, childhood economic/health status, marital status, educational level, income, smoking, drinking, body mass index, depression, hypertension, dyslipidemia, and diabetes

^b^ Stabilized weights accounted for age, sex, ethnicity, type of residence, childhood economic/health status, marital status, educational level, income, smoking, drinking, body mass index, hypertension, dyslipidemia, and diabetes

*p < .05

The percentage of the total effect of famine exposure mediated by each mediator is presented in Table 3. When all mediators were considered at the same time, cognitive function and depression accounted for a greater part of the effect for childhood famine exposure than for the two other famine exposure groups, mediating 36.35% (95% CI = 14.19–96.19%) of the overall association between childhood famine exposure and incident stroke. Depression is the stronger of these two mediators between famine exposure and risk stroke in adults. In terms of percentage mediated, depression mediated 30.36% of the overall effect between childhood famine exposure and risk of stroke and 27.55% of the association between adolescence/adulthood famine exposure with risk of stroke. However, the indirect effects through cognitive function and depression were nonsignificant for fetal-stage exposure to famine.

**Table 3:** Percentages of the Association between Early-life Famine Exposure and Incident Adult Stroke Mediated by Cognitive Function and Depression in Adulthood in CHARLS, 2011–2015

Mediators	Proportion of the natural indirect effect explained ^a^					
	Fetally exposed		Childhood-exposed		Adolescence/adulthood-exposed	
	%	95% CI	%	95% CI	%	95% CI
Cognitive function (M_1_) ^b^	2.43^*^	0.65, 5.67	6.00^*^	2.05, 16.91	5.42^*^	1.80, 10.15
Depression (M_2_) ^c^	12.96	−3.37, 45.16	30.36^*^	9.87, 87.44	22.13^*^	10.00, 35.60
M_1_ + M_2_^d^	15.38	−0.85, 48.75	36.35^*^	14.19, 96.19	27.55^*^	14.92, 40.02

*CI* confidence interval

^a^ Calculated as [natural indirect effect/(natural direct effect + natural indirect effect)]*100%

^b^ Stabilized weights accounted for age, sex, ethnicity, type of residence, childhood economic/health status, marital status, educational level, income, smoking, drinking, body mass index, depression, hypertension, dyslipidemia, and diabetes

^c^ Stabilized weights accounted for age, sex, ethnicity, type of residence, childhood economic/health status, marital status, educational level, income, smoking, drinking, body mass index, cognitive function, hypertension, dyslipidemia, and diabetes

^d^ Stabilized weights accounted for age, sex, ethnicity, type of residence, childhood economic/health status, marital status, educational level, income, smoking, drinking, body mass index, hypertension, dyslipidemia, and diabetes

*p < .05

**Fig. 2:**
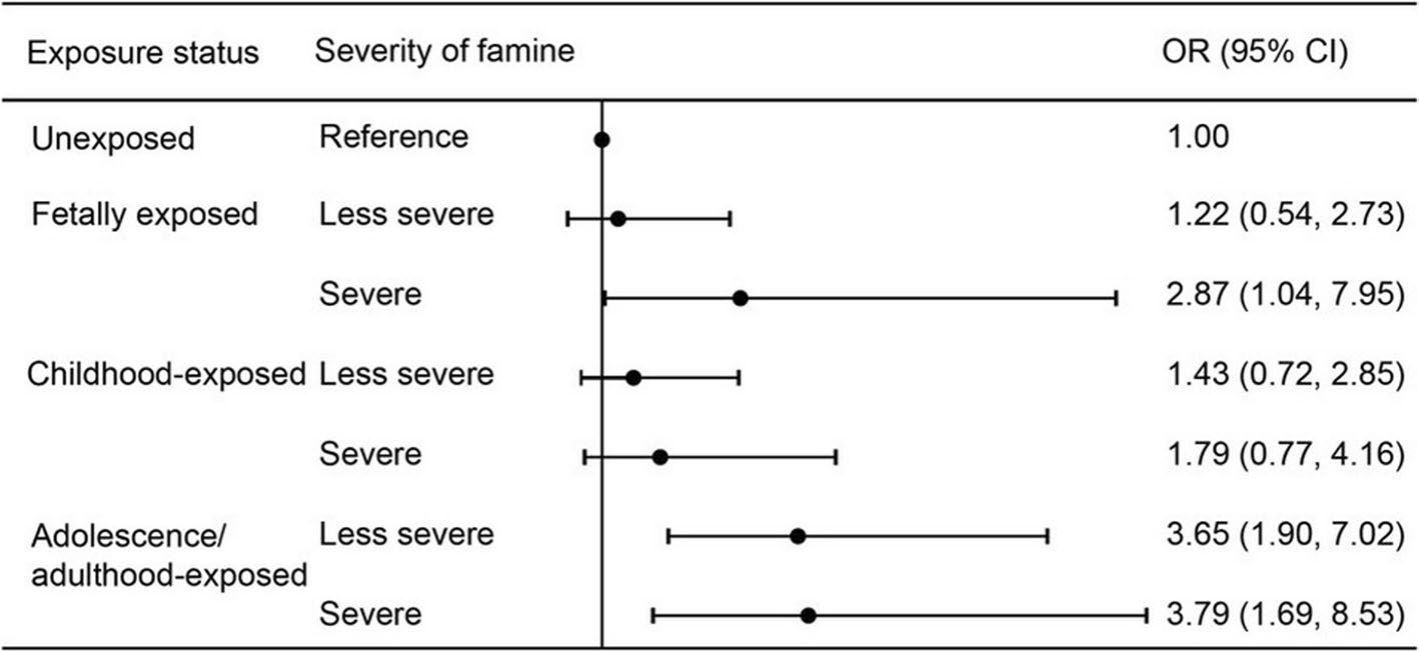
Joint Analysis of Famine Exposure and Famine Severity in Relation to the Stroke Risk in CHARLS, 2011–2015

We further classified the 12,681 participants exposed to famine according to the joint categories of exposure status and severity of famine, with subjects unexposed to famine as the reference group (Fig. 2). A dose-response relationship existed between fetal or adolescent/adult famine exposure and stroke incidence (*P* for trend< 0.05). Compared with the reference group, severe famine exposure in the fetal period (Odds ratio (OR) = 2.87, 95% CI = 1.04, 7.95) was associated with a magnified increase in stroke risk relative to less-severe famine exposure. A similar tendency between the severity of famine and stroke risk was observed among participants who were exposed to famine during adolescence/adulthood, with adjusted ORs of 3.79 (95% CI = 1.69, 8.53) for severe famine and 3.65 (95% CI = 1.90, 7.02) for less-severe famine, but not among those exposed during childhood. In general, the risk of stroke was positively correlated with the severity of famine during fetal development and adolescence/adulthood.

In sensitivity analyses, we investigated the effects of truncated weights and unmeasured confounding. When different weight truncation values were used, the associations between famine exposure and stroke through cognitive function and depression remained very similar (see supplementary Table 1). Moreover, the results of sensitivity analyses hypothesizing the presence of *U*, an unmeasured binary common cause of famine exposure and stroke, are presented in supplementary Table 2. The results of NDE and NIE are relatively stable.

## Discussion

This study applied a counterfactual framework to estimate the NDE and NIE of early famine exposure on stroke risk by using MSM for mediation analysis. In population research, the Great Chinese Famine offers us a natural chance to test this relationship. Our study, using nationally representative longitudinal study data in China, found that exposure to famine at different stages was associated with stroke risk among Chinese adults aged 45 years or more. The links between famine exposure in childhood or during adolescence/adulthood and stroke in mid- to late life are partly mediated by cognitive function and depression in adulthood after an MSM was fitted. However, the association between prenatal exposure to famine and stroke is partly mediated by cognitive function in adults but not by depression.

Our finding that famine exposure in different stages is significantly associated with higher risk stroke in later life. Moreover, we found that this relationship was dose-dependent. Compared with participants who were exposed to less-severe famine, severe famine exposure in the fetal period and adolescence/adulthood was associated with a higher future risk of stroke relative. Several direct mechanisms can explain why early-life famine exposure can increase the risk of stroke in adulthood. Animal experiments show that malnutrition in early life is closely related to arterial wall reconstruction and increased arterial stiffness in later life [30]. Furthermore, stress from famine may activate the hypothalamic-pituitary-adrenal (HPA) axis and result in excess glucocorticoid [42, 43], which may lead to stroke occurrence. This finding contrasts with findings in Dutch famine studies, which did not find associations between prenatal exposure to famine and stroke onset in adulthood and found that exposure to famine during postnatal life was associated with a reduced risk of stroke [14, 15]. The discrepancy between their research and ours may be due to methodological challenges. In the conventional model, simply adjusting for these mediators and confounders would block the link between early famine exposure and stroke, which could lead to bias. In contrast to conventional regression methods, we addressed the selection bias caused by time-varying covariates by fitting an MSM with IPTW. This method has many benefits over traditional methods; in particular, it can avoid conditioning on endogenous confounders via IPTW to obtain the NDE and NIE. We used sensitivity analyses to estimate NDE and NIE for the biases from famine–stroke, mediator–stroke and unmeasured mediator-outcome confounding. Additionally, our research focused on the effects of famine exposure in different periods, from fetal development through early adulthood, on stroke risk and adjusted the sample censoring and sampling weight to mitigate the issue of sample attrition; the findings are generalizable to the entire population.

A further important finding of this study is that cognitive function and depression partially mediated the relationship between early famine exposure and stroke in middle and older adults using MSM. The proportion of the effect mediated by depression was higher than the proportion mediated by cognitive function. One potential pathway from early famine exposure to stroke risk in adulthood is through the low cognitive function of famine exposure. Previous research has shown that early-life famine affects the human central nervous system, including the brain, and has a long-term negative impact on cognitive function in later life [9, 22]. In addition, evidence has shown that people with cognitive decline tend to have brain vascular pathologies and disturbances in cerebrovascular hemodynamics [44], which increase the long-term risk of stroke [45]. On the other hand, the stress of early famine exposure may also produce psychological changes such as depression; depression has been associated with inflammation and short-term increases in systolic blood pressure [46, 47], which, in turn, may increase stroke risk [48]. For fetuses, this may be because psychosocial skills are not developed at this stage, and they are less likely to have felt the psychological pressure of the Great Chinese Famine, which may explain the modest mediating effect of depression. Future studies are urgently necessity to confirm our finding and delineate potential mechanisms.

Estimates from the MSM relied on the assumptions for the model. First, there should be “no unmeasured confounders” of exposure–outcome, mediator–outcome or exposure-mediator paths. The results of our sensitivity analysis indicated that the unmeasured confounding U did not change substantially in the estimates. Most of the sensitivity parameters hypothesized in supplementary Table 2 that the NDE and NIE of early famine exposure on stroke were not altered; hence, we can suggest that early experience of famine is likely to influence the risk of stroke in adulthood. Second, the assumption, which requires that no variable is affected by exposure, confuses any mediator-outcome relationship, with no causal effect between mediators. When we successively introduced the mediators into the models, our estimates for the NIE of each mediator were stable, which indicates that our models are valid and that the two mediators under investigation represent different, non-intertwined causal pathways. Third, assumptions, such as positivity and correct model specification, are required for weight estimation, and a necessary condition is that the sample weights (SWs) have a mean of one. Because the means of SWs in this study tend to 1, the abovementioned assumptions can be considered valid. In addition, a tradeoff between confounding bias and variance may be encountered in making inferences with a lack of positivity. We used weight truncation to address this bias-variance tradeoff.

The current study has some unavoidable limitations that must be acknowledged. First, we lack individual data on famine exposure. Moreover, there was no exact beginning or ending date for the Great Chinese Famine. Misclassification of exposure may exist, which could lead to bias. Nevertheless, famine exposure was defined by birthdate in this study, which was consistent with previous research suggesting that this method of classification is sufficient [10, 49]. Second, the onset of stroke was mainly self-reported, which may lead to some recall bias. However, the CHARLS design was based on the HRS, and previous studies have shown that in the HRS, self-reported information is highly consistent with clinical diagnosis in the incidence of stroke [50]. Furthermore, given the lack of detailed information about stroke classification and the time of stroke onset in CHARLS, our inability to distinguish the effect on stroke subtype (ischemic stroke and hemorrhagic stroke) and to estimate the causal effect on the time to event outcome. Third, there may be other pathways, such as socioeconomic or biological factors, that may mediate the link between early famine exposure and stroke risk in adulthood [51, 52]. Due to the limitation of current data, the influence of other mediation paths should be considered in future studies. Fourth, the effects of Pack-years of cigarette smoking and alcohol intake, which take the amount and duration into account, were not examined because this information was not available in the CHARLS dataset.

## Conclusions

Despite these limitations, this study provides new application support for the three potential pathways in life-course research. After controlling for potential confounders, famine exposure during the fetal, childhood and adolescent/adult stages was related to stroke risk in middle-aged and elderly adults in China, which supports the notion that early-life nutrition is critical for the prevention and control of stroke risk in mid- to late life. Moreover, the link between early famine exposure and stroke in adults is mediated in part by cognitive function and depression. Thus, strengthening psychological support for people with early-life famine experience and elevating their cognitive function in middle and later life could undo the disadvantages accumulated over the lifespan to some extent, help to improve the quality of life of elderly people in general, and reduce health care expenditures.

## Supplementary Information

**Additional file 1: Supplementary Table 1.** Results from Marginal Structural Model Analysis of Different Truncated Weights on the Estimated Natural Direct and Indirect Effect of Famine Exposure Mediated by Cognitive Function and Depression Combined on Stroke in CHARLS, 2011-2015. **Supplementary Table 2.** Sensitivity Analysis for Unmeasured confounding (*U*) Considering Estimates of Natural Direct and Indirect Effect of Early-life Famine Exposure, Cognitive Function and Depression Combined and Stroke in CHARLS, 2011-2015 ^a^.

## Data Availability

The CHARLS dataset is publicly available. Information about the data source and available data are found at http://charls.pku.edu.cn/pages/data/111/zh-cn.html. Researchers can obtain these data after submitting a data use agreement to the CHARLS team.

## Abbreviations

CHARLS: China Health and Retirement Longitudinal Study
MSM: Marginal Structural Model
OR: Odds ratio
CI: Confidence interval
NDE: Natural direct effect
NIE: Natural indirect effect
