# Supplementary material for: Early-life exposure to Chinese famine and stroke risk in mid- to late life: the mediating roles of cognitive function and depression
Source: BMC Geriatr. 2022 Apr 7;22:294. doi: 10.1186/s12877-022-02990-z (PMC8988351; doi:10.1186/s12877-022-02990-z)
Supplement: Supplementary file 1 — Additional file 1: Supplementary Table 1. Results from Marginal Structural Model Analysis of Different Truncated Weights on the Estimated Natural Direct and Indirect Effect of Famine Exposure Mediated by Cognitive Function and Depression Combined on Stroke in CHARLS, 2011-2015. Supplementary Table 2. Sensitivity Analysis for Unmeasured confounding (U) Considering Estimates of Natural Direct and Indirect Effect of Early-life Famine Exposure, Cognitive Function and Depression Combined and Stroke in CHARLS, 2011-2015 a. [file 12877_2022_2990_MOESM1_ESM.docx]

**Appendix**

**Supplementary Table 1.** Results from Marginal Structural Model Analysis of Different Truncated Weights on the Estimated Natural Direct and Indirect Effect of Famine Exposure Mediated by Cognitive Function and Depression Combined on Stroke in CHARLS, 2011-2015

|  | 5th/95th^a^ | |  | 10th/90th^a^ | |
| --- | --- | --- | --- | --- | --- |
|  | Natural direct effect  *β* (95% CI) | Natural indirect effect  *β* (95% CI) |  | Natural direct effect  *β* (95% CI) | Natural indirect effect  *β* (95% CI) |
| Fetally exposed vs  Unexposed | 0.76 (0.04,1.64) | 0.11 (-0.02,0.21) |  | 0.69 (0.10,1.44) | 0.09 (-0.04,0.22) |
| Childhood-exposed vs Unexposed | 0.54 (0.04,1.12) | 0.28 (0.16,0.44) |  | 0.48 (0.00,1.20) | 0.25 (0.13,0.41) |
| Adolescence/adulthood-exposed vs Unexposed | 1.36 (0.75,2.00) | 0.48 (0.28,0.69) |  | 1.26 (0.79,1.94) | 0.44 (0.26,0.67) |

^a^ Stabilized weights accounted for age, sex, ethnicity, type of residence, childhood economic/health status, marital status, educational level, income, smoking, drinking, body mass index, hypertension, dyslipidemia, and diabetes.

**Supplementary Table 2.** Sensitivity Analysis for Unmeasured confounding (*U*) Considering Estimates of Natural Direct and Indirect Effect of Early-life Famine Exposure, Cognitive Function and Depression Combined and Stroke in CHARLS, 2011-2015 ^a^

| *g* | *d* | Fetally exposed vs  Unexposed | |  | Childhood-exposed vs Unexposed | |  | Adolescence/adulthood-exposed vs Unexposed | |
| --- | --- | --- | --- | --- | --- | --- | --- | --- | --- |
|  |  | Natural direct effect  *β* (95% CI) | Natural indirect effect  *β* (95% CI) |  | Natural direct effect  *β* (95% CI) | Natural indirect effect  *β* (95% CI) |  | Natural  direct effect  *β* (95% CI) | Natural indirect effect  *β* (95% CI) |
| 0.4 | 0.3 | 0.64 (0.14,1.48) | 0.02 (-0.11,0.22) |  | 0.46 (-0.10,1.08) | 0.21 (0.07,0.44) |  | 1.33 (0.88,2.04) | 0.43 (0.2,0.69) |
| 0.4 | 0.2 | 0.68 (0.18,1.52) | 0.06 (-0.07,0.26) |  | 0.50 (-0.06,1.12) | 0.25 (0.11,0.48) |  | 1.37 (0.92,2.08) | 0.47 (0.24,0.73) |
| 0.4 | 0.1 | 0.72 (0.22,1.56) | 0.10 (-0.03,0.30) |  | 0.54 (-0.02,1.16) | 0.29 (0.15,0.52) |  | 1.41 (0.96,2.12) | 0.51 (0.28,0.77) |
| 0.2 | 0.3 | 0.70 (0.20,1.54) | 0.08 (-0.05,0.28) |  | 0.52 (-0.04,1.14) | 0.27 (0.13,0.50) |  | 1.39 (0.94,2.10) | 0.49 (0.26,0.75) |
| 0.2 | 0.2 | 0.72 (0.22,1.56) | 0.10 (-0.03,0.30) |  | 0.54 (-0.02,1.16) | 0.29 (0.15,0.52) |  | 1.41 (0.96,2.12) | 0.51 (0.28,0.77) |
| 0.2 | 0.1 | 0.74 (0.24,1.58) | 0.12 (-0.01,0.32) |  | 0.56 (0.00,1.18) | 0.31 (0.17,0.54) |  | 1.43 (0.98,2.14) | 0.53 (0.30,0.79) |
| 0.1 | 0.3 | 0.73 (0.23,1.57) | 0.11 (-0.02,0.31) |  | 0.55 (-0.01,1.17) | 0.30 (0.16,0.53) |  | 1.42 (0.97,2.13) | 0.52 (0.29,0.78) |
| 0.1 | 0.2 | 0.74 (0.24,1.58) | 0.12 (-0.01,0.32) |  | 0.56 (0.00,1.18) | 0.31 (0.17,0.54) |  | 1.43 (0.98,2.14) | 0.53 (0.30,0.79) |
| 0.1 | 0.1 | 0.75 (0.25,1.59) | 0.13 (0.00,0.33) |  | 0.57 (0.01,1.19) | 0.32 (0.18,0.55) |  | 1.44 (0.99,2.15) | 0.54 (0.31,0.80) |
| -0.1 | 0.3 | 0.79 (0.29,1.63) | 0.17 (0.04,0.37) |  | 0.61 (0.05,1.23) | 0.36 (0.22,0.59) |  | 1.48 (1.03,2.19) | 0.58 (0.35,0.84) |
| -0.1 | 0.2 | 0.78 (0.28,1.62) | 0.16 (0.03,0.36) |  | 0.60 (0.04,1.22) | 0.35 (0.21,0.58) |  | 1.47 (1.02,2.18) | 0.57 (0.34,0.83) |
| -0.1 | 0.1 | 0.77 (0.27,1.61) | 0.15 (0.02,0.35) |  | 0.59 (0.03,1.21) | 0.34 (0.20,0.57) |  | 1.46 (1.01,2.17) | 0.56 (0.33,0.82) |
| -0.2 | 0.3 | 0.82 (0.32,1.66) | 0.20 (0.07,0.40) |  | 0.64 (0.08,1.26) | 0.39 (0.25,0.62) |  | 1.51 (1.06,2.22) | 0.61 (0.38,0.87) |
| -0.2 | 0.2 | 0.80 (0.03,1.64) | 0.18 (0.05,0.38) |  | 0.62 (0.06,1.24) | 0.37 (0.23,0.60) |  | 1.49 (1.04,2.20) | 0.59 (0.36,0.85) |
| -0.2 | 0.1 | 0.78 (0.28,1.62) | 0.16 (0.03,0.36) |  | 0.60 (0.04,1.22) | 0.35 (0.21,0.58) |  | 1.47 (1.02,2.18) | 0.57 (0.34,0.83) |
| -0.4 | 0.3 | 0.88 (0.38,1.72) | 0.26 (0.13,0.46) |  | 0.70 (0.14,1.32) | 0.45 (0.31,0.68) |  | 1.57 (1.12,2.28) | 0.67 (0.44,0.93) |
| -0.4 | 0.2 | 0.84 (0.34,1.68) | 0.22 (0.09,0.42) |  | 0.66 (0.10,1.28) | 0.41 (0.27,0.64) |  | 1.53 (1.08,2.24) | 0.63 (0.40,0.89) |
| -0.4 | 0.1 | 0.80 (0.30,1.64) | 0.18 (0.05,0.38) |  | 0.62 (0.06,1.24) | 0.37 (0.23,0.60) |  | 1.49 (1.04,2.20) | 0.59 (0.36,0.85) |

^a^ For this analysis, point estimates (*β*) and 95% confidence intervals (CIs) were adjusted for a hypothetical, unmeasured binary confounder by subtracting a product of 2 bias parameters: d, indicating the imbalance of the confounder between strata of the exposure, and g, indicating the effect of the confounder on the outcome.

**Marginal Structural Model (MSM) Analysis**

We estimated the weights of exposure and mediators separately and then multiplied them to obtain the final weight.

$SW_{i}^{T}=\frac{P(T=t_{i})}{P(T=t_{i}|V=v_{i})}$ (1)

and

$SW_{i}^{M}=\frac{P(M=m_{i}|T=t_{i})}{P(M=m_{i}|T=t_{i},V=v_{i},L=l_{i})}$ (2)

Here, the variable T refers to early-life famine exposure, M represents adult cognitive function and depression as mediators, V represents time-constant covariates and L represents time-varying covariates. To improve the stability of the results, we truncated the final weight at the top percentile and bottom percentile (1^st^ and 99^th^) to reduce the unreasonable inﬂuence of extreme weights on the NDE and NIE.

To adjust for censoring, we calculated an inverse-probability-of-censoring weight in MSM. Specifically, let C=0 if a subject was uncensored and completed follow-up.

$SW_{i}^{C}=\frac{P(C=0|T=t_{i})}{P(C=0|T=t_{i},V=v_{i},L=l_{i})}$ (3)

Second, we used 2 MSMs to estimate the NDE and NIE. Figure 1 shows a hypothetical relationship between famine exposure and stroke risk.

$logit p\left(Y=1|T=t,M=m,V=v\right)=\theta_{0}+\theta_{1}T+\theta_{2}M+\theta_{3}V$ (4)

$logit p\left(M=1|T=t,V=v\right)=\beta_{0}+\beta_{1}T+\beta_{2}V$ (5)

Under the assumption that there was not a causal relationship between the two mediators, we did not include their correlation in the analysis. We used the weight of each observer as $SW_{tm}\text{=}SW_{i}^{T}\times SW_{i}^{M}\times SW_{i}^{C}\times SW_{i}^{S}$ for equation 4, and the weight of $SW_{t}=SW_{i}^{T}\times SW_{i}^{C}\times SW_{i}^{S}$ for equation 5, to estimate the NDE and NIE. The NDE and NIE are given by $E\left[Y_{tM_{t\text{*}}}-Y_{t\text{*}M_{t\text{*}}}|l\right]=\theta_{1}(t-t\text{*})$ and $E\left[Y_{tM_{t\text{*}}}-Y_{tM_{t\text{*}}}|l\right]=\theta_{2}\beta_{1}(t-t\text{*})$, respectively. The total effect can be divided into NDE and NIE. Confidence intervals (CI) based on 5000 replications were calculated using a bootstrap resampling method.

**Stata codes to create the dataset**

* using data from Wave 1 (2011) of CHARLS

use demographic_background.dta, clear

merge 1:1 ID using health_status_and_functioning.dta

merge 1:1 ID using individual_income.dta, generate

merge 1:1 ID using health_care_and_insurance.dta, generate

merge 1:1 ID using biomarker.dta, generate

save data_2011.dta, replace

* using data from Wave 2 (2013) of CHARLS

use demographic_background.dta, clear

merge 1:1 ID using health_status_and_functioning.dta

merge 1:1 ID using individual_income.dta, generate

merge 1:1 ID using health_care_and_insurance.dta, generate

merge 1:1 ID using biomarker.dta, generate

save data_2013.dta, replace

* using data from Wave 3 (2014) of CHARLS

use Family_Information.dta, clear

merge 1:1 ID using Health_History.dta, generate

save data_2014.dta, replace

* using data from Wave 4 (2015) of CHARLS

use Health_Status_and_Functioning.dta, clear

save data_2015.dta, replace

* The dataset was created using data from Wave 1 through Wave 4 of CHARLS

merge 1:1 ID using data_2011.dta, generate

merge 1:1 ID using data_2013.dta, generate

merge 1:1 ID using data_2014.dta, generate

save final_data.dta, replace
